# Placenta-Driven Evolution: Viral Gene Acquisition and *PEG10*’s Essential Roles in Eutherian Placenta

**DOI:** 10.3390/biom16010161

**Published:** 2026-01-16

**Authors:** Hirosuke Shiura, Moe Kitazawa, Tomoko Kaneko-Ishino, Fumitoshi Ishino

**Affiliations:** 1 Faculty of Life and Environmental Sciences, University of Yamanashi, Kofu 400-8510, Japan; hshiura@yamanashi.ac.jp (H.S.); moe@yamanashi.ac.jp (M.K.); 2 School of Medicine, Tokai University, Isehara 259-1193, Japan; 3 Research Infrastructure Management Organization, Institute of Science Tokyo (IST), Tokyo 113-8501, Japan

**Keywords:** placenta-driven evolution, viral gene acquisition, *PEG10*, *PEG11/RTL1*, SIRH/RTL genes, extraembryonic tissues, placenta, yolk sac, brain, neuron, microglia

## Abstract

Mammalian placentation represents one of the most striking evolutionary innovations among vertebrates, and accumulating evidence indicates that virus-derived genes—particularly the metavirus-derived *PEG10* and *PEG11/RTL1*—have played indispensable but distinct roles: *PEG10* in the emergence of therian viviparity and *PEG11/RTL1* in the subsequent differentiation between marsupial and eutherian placental types. Notably, the metavirus-derived SIRH/RTL gene group, which includes *PEG10* and *PEG11/RTL1*, exhibits unique and diverse functions not only in placenta development but also within microglia of the brain. Because microglia originate from yolk sac progenitors, these findings suggest that extraembryonic tissues such as the placenta and yolk sac provided permissive environments that enabled the retention, expression and functional domestication of virus-derived sequences. Once the placenta itself was established through viral gene integration, it may in turn have acted as a powerful driver of eutherian evolution through recurrent acquisition and co-option of additional virus-derived genes—a process we refer to as **“placenta-driven evolution.”** This perspective offers a unified framework in which viral gene acquisition is viewed as a key driver of genomic innovation, tightly intertwined with the emergence of viviparity, subsequent divergence at the marsupial–eutherian split, and continued diversification of placental structure and function across eutherian lineages.

## 1. Introduction

The emergence of the placenta was one of the most consequential events in mammalian evolution, enabling therian mammals to give birth to live young through viviparity [1,2,3,4]. How did this remarkable organ originate? Placental evolution likely reflects a synthesis of *emergent* processes—novel interactions among pre-existing biological components, such as fetal membranes and resident host genes—and *innovative* processes driven by the acquisition of new genes.

**Figure 1:**
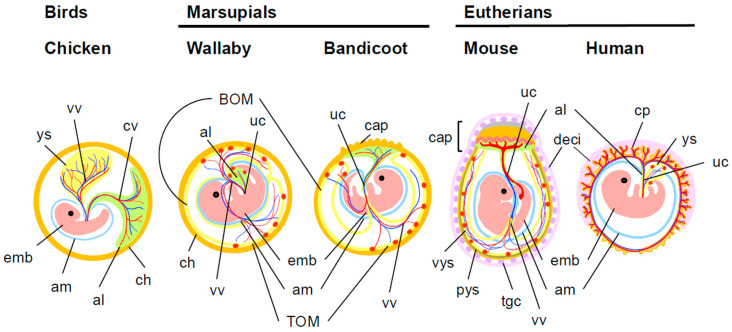
Schematic representation of the fetal membranes in birds, marsupials and eutherians: chicken (middle stage), tammar wallaby and bandicoot (late gestation), mouse (middle gestation) and human (end of 2^nd^ months of pregnancy). Note that the red vessels in the umbilical cord and yolk sac are arteries that carry deoxygenated blood from the fetus to the placenta or yolk sac, respectively. In contrast, the blue vessels represent veins that return oxygen- and nutrient-rich blood to the developing fetus. al: allantois; am: amnion; BOM: bilaminar omphalopleure; cap: chorioallantoic placenta; ch: chorion; cp: chorinic plate; deci: decidua; emb: embryo; pys: parietal yolk sac; tgc: trophoblast giant cell; TOM: trilaminar omphalopleure; uc: umbilical cord; vys: visceral yolk sac; vv: vitelline vessel; ys: yolk sac. The diagrams of extraembryonic tissues were referenced from the following papers: chicken [5,6,7], wallaby [3,4,8,9,10,11], bandicoot [8], mouse [12], human [2,13].

In eutherians, the placenta arises through fusion of the allantois and chorion (chorioallantoic placenta)—two fetal membranes conserved across amniotes (reptiles, birds, and mammals) [1,2]. In contrast, marsupial yolk sac placenta is formed through interactions between a different combination of two fetal membranes, the yolk sac and the chorion (choriovitelline placenta) [3,4]. Although these three fetal membranes are ancestrally shared among amniotes, their distinct combinations in therian mammals give rise to a fundamentally new organ with specialized and highly diversified biological functions—the placenta (Figure 1).

Large-scale knockout (KO) mouse studies have shown that placental abnormalities are extremely prevalent among embryonic lethal mutants [14,15,16]. These studies suggest that several thousand genes are required for early mammalian development [2,17], and because placental defects are observed in the majority of embryonic lethal mutants, it is likely that well over a thousand genes contribute, directly or indirectly, to placental development and function. In addition, a growing body of evidence indicates that a small number of virus-derived genes have made disproportionately important contributions to this evolutionary transformation, with *PEG10* playing a particularly central role in the emergence of placentation, and *PEG11/RTL1* serving as a key factor in establishing the eutherian-type of chorioallantoic placenta [18,19,20]. In the mouse placenta, at least five virus-derived genes are known to be essential for its formation, function, and maintenance [20]. Thus, the origin of the placenta depended on the acquisition of genes from external sources—primarily metaviruses and retroviruses—and the domestication of their molecular machinery. In this sense, viral gene acquisition represents a key evolutionary event underlying the establishment of viviparity in eutherian mammals. Considering how such genes were likely acquired raises an intriguing possibility.

In this review, we summarize the essential roles of virus-derived genes in placental development and evolution, explore the mechanisms by which such genes are acquired and domesticated, and propose a conceptual framework for understanding mammalian genomic innovation—**the “placenta-driven evolution” hypothesis**, which describes how subsequent evolutionary trajectories were shaped by the presence of the placenta through the recurrent acquisition and domestication of such virus-derived genes.

## 2. Results

### 2.1. Placental Development and Evolution: Five Virus-Derived Genes Essential for the Eutherian Placenta

**Figure 2:**
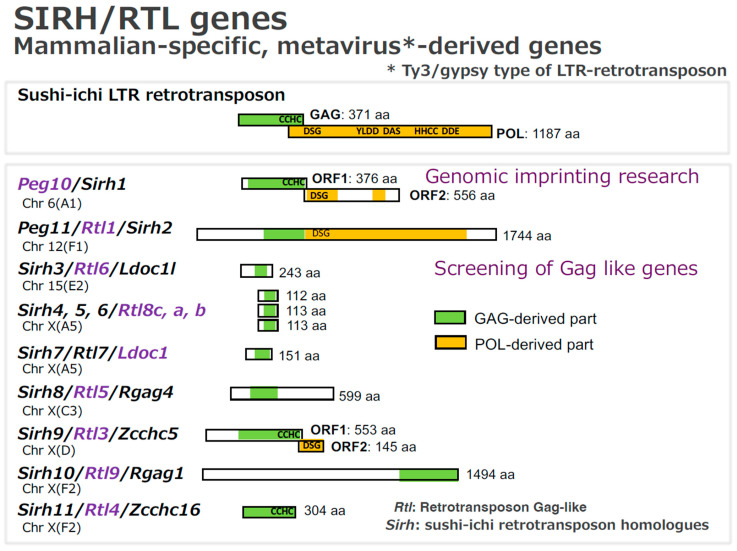
Unique and diverse SIRH/RTL proteins in eutherians: This figure shows mouse SIRH/RTL proteins, and their chromosomal locations. CCHC: RNA-binding motif; DAS: RNase highly conserved motif; DDE: strongly conserved integrase motif; DSG: aspartic protease active site; HHCC: integrase DNA-binding motif; YLDD: reverse transcriptase motif. Official names are shown in purple.

Eutherian mammals possess eleven sushi-ichi retrotransposon homolog (SIRH)/retrotransposon Gag-like (RTL) genes derived from metaviruses, the formerly classified as Ty3/gypsy LTR retrotransposons in their genomes [18,19,20,21,22,23,24,25,26,27,28,29,30] (Figure 2). Among these, at least three SIRH/RTL genes are known to play essential roles in placental formation, maintenance and functions, together with two *syncytin* genes—retrovirus-derived envelope (*ENV*) genes that arose uniquely in the eutherian lineage [31,32,33,34,35].

All SIRH/RTL genes encode proteins exhibiting homology to the GAG of the sushi-ichi LTR retrotransposon. However, each protein has a distinct amino acid sequence and differs markedly in protein length, resulting in unique and functionally divergent biological roles. Among them, PEG10, PEG11/RTL1 and SIRH9/RTL4 retain POL-derived domains. Evolutionary conservation patterns also differ among the SIRH/RTL genes. *PEG10* is therian-specific and conserved in both marsupials and eutherians, whereas *PEG11/RTL1* and the remaining SIRH/RTL genes are eutherian-specific. Among eutherians, *PEG10*, *PEG11/RTL1*, *SIRH3/RTL6*, *SIRH4, 5, 6/RTL8c, a, b* and *LDOC1/SIRH7* are conserved across all lineages examined. By contrast, *SIRH8/RTL5*, *SIRH9/RTL4*, *SIRH10/RTL9* and *SIRH11/RTL4* show lineage-specific degeneration or gene loss in certain species.

#### 2.1.1. Therian-Specific, Metavirus-Derived *PEG10* Has Multiple, Stage-Specific, Essential Roles in the Placenta

**Figure 3:**
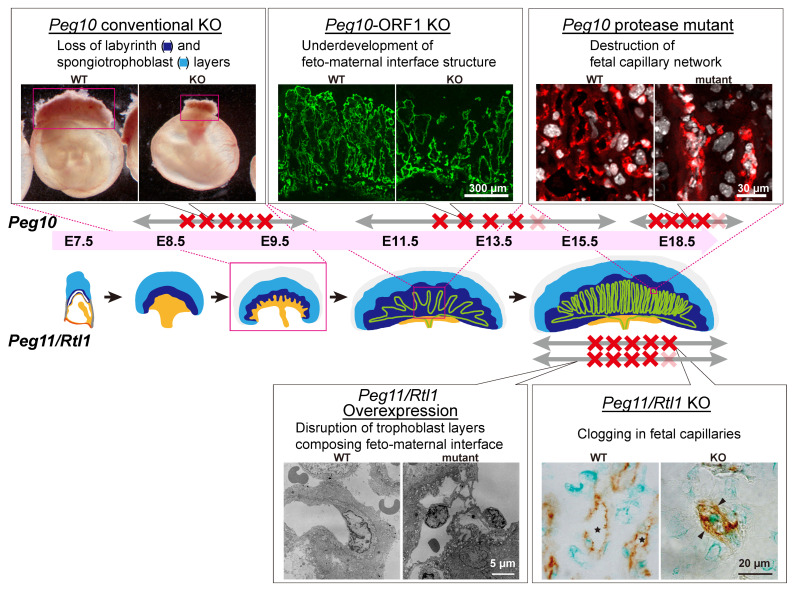
Essential roles of PEG10 and PEG11/RTL proteins in placenta development. **Top**: Multiple essential and stage-specific roles of PEG10 proteins/fragments. **Bottom**: Abnormal fetal capillaries under overexpression and deficiency of PEG11/RTL1 protein. The red X indicates the period when fetal death was confirmed. The red box of E9.5 indicates that the WT and *Peg10* KO conceptuses are obtained at the same stage. The loss of the maternal anti-*Peg11/Rtl1* expression containing several miRNAs that target *Peg11/Rtl1* mRNA results in the overexpression of *Peg11/Rtl1* [36,37,38,39].

*PEG10* is a therian-specific gene conserved in both marsupials and eutherians, representing the earliest metavirus-derived gene integrated during mammalian evolution [23,24,25,26,27,28]. PEG10 proteins retain clear homology with the GAG and POL proteins of the sushi-ichi LTR retrotransposon [23,24,25]. However, the protein similarity between PEG10 and the ancestral GAG/POL proteins is only approximately 20–30%, indicating that a substantial number of mutations must have accumulated following the original metaviral insertion before *PEG10* became functionally established [24]. We refer to this type of domesticated gene as an “acquired gene”. *Peg10*–null mice exhibit early embryonic lethality due to the complete absence of two major trophoblast-derived structures—the labyrinth layer and spongiotrophoblast layer [28] (Figure 3, top left). Because trophoblasts are unique to the mammalian placenta [1], these findings demonstrate that *PEG10* is indispensable not only for trophoblast lineage specification at the earliest stages of placental development but also for the establishment of viviparity itself.

Recent studies reveal that *PEG10* performs multiple essential and stage-specific roles through its complex gene structure, which retains several characteristics of its metavirus origin. *PEG10* encodes two proteins—PEG10-ORF1 and a PEG10-ORF1/ORF2 fusion protein—generated through a − 1 ribosomal frameshift mechanism conserved across retroviruses and metaviruses [23,24,25,40,41,42,43]. Both proteins are further processed by an intrinsic DSG protease, producing multiple cleaved fragments. Remarkably, all these features are conserved across therian mammals, strongly suggesting that each protein—and potentially each processed fragment—fulfills distinct roles during placental morphogenesis.

Consistent with these characteristics, PEG10-ORF1 KO mice exhibit mid- to late-gestational lethality [44], in contrast to *Peg10*-null mice that die much earlier. ORF1-deficient embryos display delayed development of the fetal capillary network within the labyrinth layer. At embryonic day 10.5 (E10.5), clusters of undifferentiated trophoblast cells persist, and the labyrinth trophoblast progenitor (LaTP) cells fail to appear at the appropriate developmental time [44,45,46]. In mature placentas, fetal capillary endothelial cells are normally surrounded by two syncytiotrophoblast layers (SynT-I and SynT-II) and sinusoidal trophoblast giant cells (sTGCs), all derived from LaTP cells [45,46]. In ORF1 mutants, this architecture is disorganized and coarse, compared with the fine and dense network seen in wild type placenta, and over 80% of embryos die before birth [44] (Figure 3, top middle).

A PEG10-ASG mutant—harboring a catalytic DSG → ASG substitution in the protease domain—exhibits yet another phenotype. Although placental morphology initially appears normal until mid-gestation, the fetal capillary network progressively collapses during late gestation, again resulting in more than 80% perinatal lethality [47] (Figure 3, top right).

Together, these findings demonstrate that *PEG10* functions as a multi-component, multi-stage regulator of placental development. Through its complex metavirus-derived architecture, *PEG10* promotes trophoblast differentiation in both the spongiotrophoblast and labyrinth layers and safeguards the fetal capillary network throughout gestation. The totality of evidence strongly supports the idea that *PEG10* acquisition represented a pivotal evolutionary event, enabling the emergence of the placenta and establishing the foundation for viviparity in therian mammals.

#### 2.1.2. Eutherian-Specific, Metavirus-Derived *PEG11/RTL1* and *LOC1/SIRH7/RTL7* Are Essential for the Establishment of the Eutherian-Type Placenta

While *PEG10* is essential for the fundamental formation of the therian placenta, two additional metavirus-derived genes—*PEG11/RTL1* and *LDOC1/SIRH7/RTL7*—are required for constructing the eutherian-type placenta that supports the prolonged gestation characteristic of eutherian mammals [20].

*PEG11/RTL1* encodes a protein homologous to sushi-ichi GAG and POL, including an active DSG protease motif similar to that of PEG10 [48,49]. Unlike *Peg 10*, however, *Peg11/Rtl1* is expressed in fetal capillary endothelial cells and adjacent SynT-II cells [36,50]. It plays a central role in maintaining the internal integrity of the fetal capillaries. Loss of *Peg11/Rtl1* results in severe late-gestational lethality caused by occlusion of fetal capillaries due to excess proliferation and invasive activity of SynT-II cells [36,51] (Figure 3, bottom right). Conversely, *Peg11/Rtl1* overexpression produces the opposite defect: excessive vacuolization and degeneration of SynT-II cells and abnormal enlargement of fetal capillary lumens, leading to fetal edema [36,51] (Figure 3, bottom left). Because *Peg 10* is also expressed in SynT-II cells [47], cooperative interactions between *Peg 10* and *Peg11/Rtl1* likely contributed to the evolutionary elaboration of the fetal capillary system required for extended gestation.

*LDOC1/SIRH7* encodes a small protein (~135 amino acids) homologous to sushi-ichi GAG [52,53]. Like *PEG10*, it is expressed in all trophoblast lineages and is essential for proper trophoblast differentiation and maturation. In *Ldoc1*/*Sirh7* knockout mice, the normal three-layered placental structure is disrupted, characterized by invasion of the spongiotrophoblast layer into the labyrinth layer [52]. In addition to structural roles, the placenta functions as a major endocrine organ, producing progesterone (P4), placental lactogens in trophoblast giant cells (TGCs), and more than 20 prolactin-related hormones in spongiotrophoblast cells [54,55,56,57]. Consistent with this endocrine function, *Ldoc1*/*Sirh7* KO mothers exhibit delayed parturition caused by impaired placental hormone regulation, particularly P4; as a result, many newborns die shortly after birth, due to abnormal maternal behavior possibly caused by delayed parturition [52]. These findings demonstrate that *LDOC1*/*SIRH7* is indispensable not only for placental architecture but also for the endocrine regulation necessary for sustained gestation and successful birth in eutherian mammals.

#### 2.1.3. Rodent-Specific, Retrovirus-Derived *Syncytin-A* and *Syncytin-B* Are Indispensable for Syncytiotrophoblast Formation

*Syncytins* have been repeatedly and independently acquired in various mammalian lineages from distinct retroviral *ENV* genes, yet they share a conserved function: mediating trophoblast cell–cell fusion to generate syncytiotrophoblasts [31,32,33,34,35,58,59]. In mice, the rodent-specific *syncytin-A* and *syncytin-B* genes fulfill this role, whereas humans and other primates possess *syncytin-1* and *syncytin-2*. Targeted disruption of *syncytin-A*—expressed in SynT-I cells—causes embryonic lethality at mid-gestation (E12–13) [58]. Combined disruption of both *syncytin-A* and *syncytin-B* leads to embryonic lethality even earlier, demonstrating that these retrovirus-derived fusogens are essential for placental development [59].

The two syncytiotrophoblast layers surrounding the fetal capillary endothelial cells form a continuous syncytial sheet through virus-derived cell fusion. This structure is thought to provide a key immunological barrier against the maternal immune system. Consistent with this view, *syncytin-B* exhibits measurable immunosuppressive activity [59,60]. Because fetal-derived cells directly contact maternal tissues during placental invasion and capillary formation, protection from maternal immunity is essential for maintaining pregnancy.

According to the “baton-passing hypothesis,” *syncytin* genes may undergo sequential replacement whenever newly invading retroviruses contribute *ENV* genes with more efficient fusogenicity [60,61]. If so, the common ancestor of therian mammals—including the lineages leading to both eutherians and marsupials—may have harbored an ancestral *syncytin* gene critical for forming a primitive placenta.

In summary, at least five virus-derived genes—*PEG10*, *PEG11/RTL1*, *LDOC1/RTL7* and two *syncytin* genes (*syncytin-A* and *syncytin-B* in mice and *syncytin-1* and *syncytin-2* in humans)—perform essential, nonredundant functions in establishing, maintaining, and elaborating the eutherian placenta. To our knowledge, the placental defects observed in *Peg10* KO, PEG10-ORF1 KO, PEG10-ASG mutant, *Peg11/Rtl1* KO, and *syncytin* KO mice are phenotypically distinct and unique, with no comparable abnormalities identified among the large and diverse collection of placental mutants reported to date [2,15,17]. This uniqueness strongly suggests that virus-derived genes have assumed specialized and nonredundant functions in placental development. Thus, their integration into mammalian genomes constituted a key evolutionary event in the origin of viviparity and subsequent diversification of eutherian mammals.

### 2.2. Roles of SIRH/RTL Genes in Yolk Sac-Derived Microglia of the Brain

In addition to their indispensable roles in the placenta, SIRH/RTL genes have also acquired essential functions in the eutherian brain (Table 1). Their roles in the neurons have been summarized elsewhere [20], including the overexpression of *PEG10* in Angelman syndrome (AS) and amyotrophic lateral sclerosis (ALS) [62,63,64,65,66], the overexpression and deficiency of *PEG11/RTL1* in Kagami–Ogata syndrome (KOS14) and Temple syndrome (TS14) [36,37,51,67,68,69,70,71], the lack of *Ldoc1/Sirh7* in abnormal maternal behavior [52], and the reduced levels of *Sirh4/5/6 (Rtl8c/a/b)* in Prader–Willi syndrome-like phenotypes [72]. These findings reveal that SIRH/RTL genes initially expressed highly in the placenta have secondarily acquired diverse and substantial functions in the nervous system [20].

Particular interest lies in the distinct subset of SIRH/RTL genes functions in microglia, the sole immune cell lineage residing within the brain. Examples of this specialization include *SIRH11/RTL4/ZCCHC16*, a causative gene for autism spectrum disorder (ASD) [73,74,75], and *SIRH3/RTL6*, *SIRH8/RTL5* and *SIRH10/RTL9*, which participate in pathogen defense [76,77], illustrate this specialization. The innate immune system—highly conserved across most animals—typically relies on Toll-like receptors that recognize generic pathogen-associated molecular patterns (PAMPs) [78,79,80]. However, our recent work shows that eutherians have elaborated this system further: these virus-derived SIRH/RTL genes enable microglia to directly recognize and eliminate specific PAMPs in a highly specialized manner, representing a striking example of molecular innovation through viral gene domestication [74,77]. Importantly, *Sirh11/Rtl4* KO mice exhibit not only abnormal behaviors—such as increased impulsivity, decreased ability to adapt to new environments, and impaired short spatial memory—but also a delayed recovery of noradrenaline levels in the prefrontal cortex [74]. Consistent with this, the SIRH11 protein appears to respond to psychological stressors, possibly by sensing changes in noradrenaline release in the brain [75].

Microglia originate from the yolk sac and migrate into the brain during early embryogenesis [81,82]. Alongside the subgroup that functions in the placenta, this developmental origin underscores that most SIRH/RTL gene subgroups retain deep ties to extraembryonic tissues. Importantly, however, their subsequent diversification has proceeded along two major trajectories—one toward neuronal functions and the other toward microglial immune functions. Together, these dual directions demonstrate that the SIRH/RTL repertoire has been repeatedly co-opted to shape both neural circuitry and innate immune specialization in the eutherian brain.

**Figure 4:**
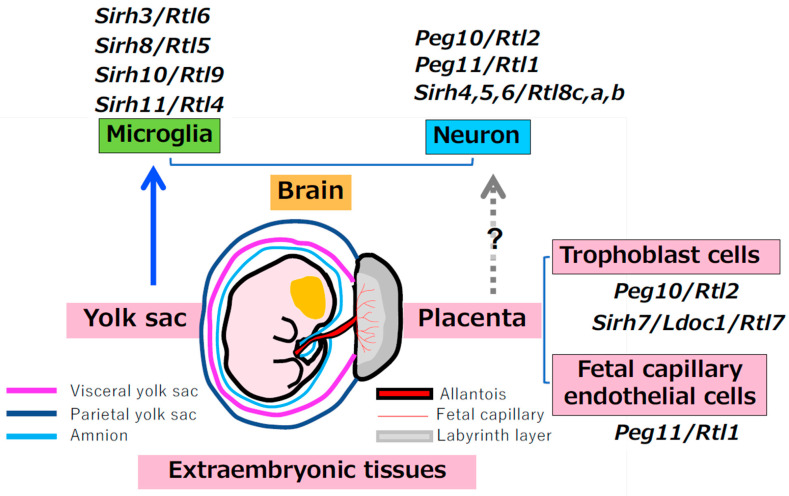
Metavirus-derived genes in extraembryonic tissues. Four SIRH/RTL genes functioning in microglia are related to the yolk sac, because microglia originate from yolk sac and migrate to the brain during early development (**Left**). Other SIRH/RTL genes and also *syncytins* function in the placenta (**right**).

Taken together, these observations strongly support the idea that extraembryonic tissues, such as the placenta and yolk sac, served as suitable “birthplaces” for the virus-derived genes [19,20,76,83] (Figure 4). This extraembryonic origin may provide the developmental and epigenetic conditions that enabled SIRH/RTL genes to be recruited not only into placental function but also into neuronal and microglial roles, thereby linking viral gene domestication to both placental and brain evolution in eutherian mammals.

### 2.3. How Were Virus-Derived Genes Acquired in Therian Mammals?—A Two-Step Evolutionary Model for the Acquisition of Viral Genes in the Placenta

**Figure 5:**
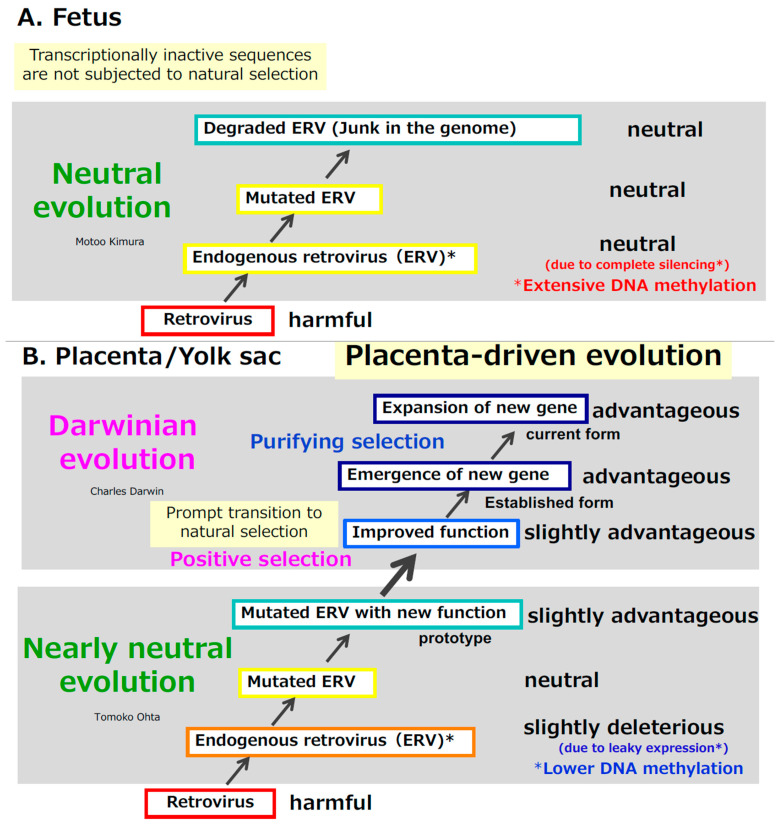
Two models of domestication of Retrovirus-derived genes. (**A**) (**top**): In the case of fetus. (**B**) (**Bottom**): In the case of extraembryonic tissues (placenta and yolk sac).

Taking retroviruses as an example, once they are integrated into the germline as endogenous retroviruses (ERVs), their expression in the fetus becomes completely silenced through extensive DNA methylation [84,85]. As a result, they behave as neutral sequences. Over time, however, transcriptionally inactive ERVs accumulate mutations and become progressively degraded, as they are not subject to natural selection (Figure 5A). In this way, any newly arising mutations in ERVs within the germline cannot be functionally tested in the fetal tissues.

In contrast, in extraembryonic tissues such as the placenta and yolk sac, ERVs exhibit leaky expression owing to substantially lower levels of DNA methylation (Figure 5B) [86,87]. This creates a unique environment in which the placenta can, in effect, “test” whether these viral elements are harmful, neutral, or potentially beneficial within a relatively protected developmental context. As mutations accumulate, some ERV sequences may shift from slightly deleterious to effectively neutral and eventually to slightly advantageous. Once a sequence acquires a slightly advantageous function, Darwinian positive selection acts to refine and optimize it. In this way, an initially foreign viral sequence can ultimately become established as a functional gene. Thereafter, purifying selection preserves and stabilizes the gene within the population (Figure 5B).

Tomoko Ohta extended Kimura’s neutral theory into the “nearly neutral theory,” proposing that slightly deleterious mutations can become fixed when the effective population size is small [88,89,90]. Our scenario fits well within this theoretical framework: leaky expression in the extraembryonic tissues would have rendered ERV-derived sequences slightly deleterious, and mammals—particularly early ancestral mammals—likely had small effective population sizes, facilitating the fixation of such sequences by genetic drift.

Thus, the gene acquisition from retroviruses can be explained by a two-step evolutionary process: an initial phase driven by genetic drift, as predicted by the nearly neutral theory, followed by Darwinian natural selection acting on sequences that acquire beneficial functions. The presence of the placenta enabled the ERV sequences to be expressed and evaluated, allowing them to accumulate multiple advantageous mutations and ultimately transform into new functional genes by acting as an “experimental laboratory” or “evolutionary testing ground” [19,20,76,83] (Figure 5B).

Importantly, tissues that maintain low levels of DNA methylation during key developmental windows—most notably the placenta, the yolk sac, and even the preimplantation embryo—constitute privileged environments for the acquisition and domestication of virus-derived genes.

### 2.4. Placenta-Driven Evolution

Extending this logic, extraembryonic tissues—particularly the placenta—may have acted as an engine of mammalian evolution, continuously accelerating genomic innovation through the recurrent acquisition of virus-derived genes. Because the placenta represents the most prominent and influential site of such events, we refer to this process as “placenta-driven evolution” (Figure 5B).

The earliest metavirus-derived gene in vertebrates is activity-regulated cytoskeleton-associated protein (*ARC*), which was acquired in the vertebrate lineage around the emergence of tetrapods (350–400 Ma) [30,91]. The second acquisition corresponds to the SCAN family genes, which arose in the common amniote ancestor (~310 Ma) [91]. Although the corresponding metavirus remains unidentified, the SCAN-GAG clearly shows homology to the Gypsy/Ty3 GAG [91]. *SASPase* (*ASPRV1*) encodes a retroviral-like aspartic protease and likely originated from an ancient retrovirus or retrovirus-derived element integrated into the mammalian genome (~168 Ma). It plays an essential role in the maintenance of the mammalian skin barrier [92,93,94]. This represents an example in which a POL-derived protease has been domesticated to fulfill an essential physiological function in mammals. Although its protease domain resembles retroviral Pol-encoded proteases, the specific retrovirus or LTR retrotransposon progenitor has not been definitively identified. Later, *PEG10* was acquired in the common therian ancestor approximately 148–166 Ma [29]. Importantly, aside from the expansion of SCAN genes through extensive gene duplication in the mammalian lineage, no other metavirus-derived genes appear to have been retained in the therian genomes between the *ARC* and *PEG10* domestication events, suggesting the gene acquisition from metavirus was extremely rare during this long interval [30].

In striking contrast, following the acquisition of *PEG10*, eutherian mammals gained ~30 metavirus-derived genes, including *PEG11/RTL1*, other SIRH/RTL genes and the paraneoplastic Ma antigen (PNMA) family genes [95,96,97,98,99,100], all of which originated after the marsupial–eutherian split (~148 Ma) [30,91]. This burst of gene acquisition raises the possibility that the emergence of the eutherian type of placenta facilitated or promoted the recurrent domestication of metavirus-derived genes. This pattern strongly supports our “placenta-driven evolution” hypothesis.

In contrast, only three metavirus-derived genes have been identified in the marsupial lineage: *PEG10* and two marsupial-specific genes, *SIRH12* [101] and *PNMA-MS1* [102]. This disparity likely reflects fundamental differences in placental biology, particularly the duration and physiological performance of the placenta. Marsupials rely primarily on a yolk sac placenta that functions only briefly during a short gestation period, whereas eutherians utilize a long-acting chorioallantoic placenta that supports sustained maternal–fetal exchange throughout extended gestation [1,2,3,4]. Such prolonged extraembryonic environments may create expanded opportunities for viral gene domestication (see Section 3).

Mammalian genomes also contain more than ten thousand ERV-derived protein-coding sequences encoding peptides longer than 80 amino acids, most of which remain uncharacterized, with the notable exception of the SIRH/RTL genes and *syncytins* [103]. Importantly, a subset of these sequences has recently been identified by several proteomics studies [104,105,106], indicating that they are indeed translated and should therefore be regarded as bona fide genes. A comparative genomics and proteomics study by Wang and Han (2020) identified 177 retrovirus-derived genes across vertebrates—including fish, amphibians, reptiles, birds, and mammals—138 of which are enriched in mammals, with a stark contrast of 136 in eutherians versus only 2 in marsupials [107]. This remarkable enrichment in mammals among vertebrates and in eutherians among therians strongly suggests that the eutherian lineage recruited viral genes at a substantially higher rate, likely facilitated by the permissive epigenetic environment of the chorioallantoic placenta. These patterns further support our “placenta-driven evolution” hypothesis.

Taken together, these observations support the view that the placenta first emerged as an essential reproductive organ through viral gene integration, and subsequently served as a powerful engine of “placenta-driven evolution” through recurrent acquisition and domestication of virus-derived genes.

## 3. Discussion

From a morphological perspective, it is notable that the eutherian chorioallantoic placenta has diversified into several structurally and functionally distinct forms—hemochorial, endotheliochorial, and epitheliochorial [108,109,110]. How such diversity arose among eutherian lineages remains an important evolutionary question. The lineage-specific acquisition of *syncytin* genes likely contributed to these differences, given their essential roles in trophoblast fusion and placental morphogenesis. However, it is almost certain that additional mechanisms—including gene duplication, modification of pre-existing genes, and changes in regulatory networks—have also played substantial roles. Furthermore, multiple retrovirus-derived sequences have been independently acquired in different eutherian lineages, and some have been shown to be protein-coding [103]. The placenta-driven evolution hypothesis aligns well with this view and further suggests that numerous yet-uncharacterized virus-derived genes may have contributed to lineage-specific diversification of placental morphology and function.

Moreover, determining which placental type characterized the last common ancestor of eutherians will provide a critical foundation for reconstructing the evolutionary trajectory of placental diversification. Phylogenetic analyses suggest that highly invasive types, such as hemochorial or endotheliochorial placentas, represent the ancestral state, whereas less invasive forms, such as epitheliochorial placentas, are derived [109,110]. This view is supported by recent studies of maternal-side uterine gene expression, which also favor a hemochorial ancestral condition [111]. Because both mice and humans possess hemochorial placentas, the phenotypes observed in knockout mice for *PEG10*, *PEG11/RTL1*, and *SIRH7/LDOC1/RTL7*—all conserved across eutherians—provide valuable insights into the developmental logic of the ancestral eutherian placenta. In this sense, these functional data offer a powerful framework for reconstructing placental evolution by linking molecular innovation to the morphological and physiological diversification of the therian as well as eutherian lineages.

Virus-derived genes appear to have disproportionately contributed to organs that underwent major evolutionary innovations in mammals, including humans, particularly the placenta and the brain. It is likely that such proteins provided pre-adapted molecular solutions to challenges unique to these organs. These observations suggest that viral gene domestication may have facilitated rapid functional diversification in evolutionarily novel tissues. In mammalian evolution, metavirus-derived SIRH/RTL genes began with the acquisition of *PEG10* in the common ancestor of therian mammals [19,20]. Recent studies have demonstrated that PEG10 performs multiple, indispensable functions during placental development. In addition to driving trophoblast proliferation and differentiation in both the spongiotrophoblast and labyrinth layers at early stages [28], PEG10-ORF1 is essential for the differentiation of syncytiotrophoblasts and sinusoidal trophoblast giant cells that envelop fetal capillaries in the labyrinth [44]. Moreover, PEG10-DSG protease is required to maintain the integrity of the fully formed fetal capillary network throughout gestation [47]. Thus, a single virus-derived gene orchestrates the formation of several fundamental placental structures and ultimately maintains the fetal capillary network—the core functional unit of the placenta. These findings underscore how critical the acquisition of *PEG10* was for the emergence of the eutherian placenta.

Although *PEG10* is present in both marsupials and eutherians, marsupials predominantly rely on a yolk sac placenta [3,4,8,20] (Figure 1). Why, then, did these two major therian lineages diverge so markedly in the type of placenta they employ? The observation that bandicoots—a marsupial species—form a chorioallantoic placenta shortly before birth, in addition to a yolk sac placenta, indicates that marsupials are not inherently incapable of producing a eutherian-type placenta [8]. Rather, this suggests that the decisive difference lies not in developmental potential but in the genetic components required to sustain prolonged placental function. A likely factor is the presence or absence of *PEG11/RTL1* [18,19,20,48,112]. Marsupial gestation periods are generally short, and in bandicoots the chorioallantoic placenta operates for only two to three days [8]. Even if such a placenta can form, the inability to maintain the fetal capillary network for an extended duration severely limits its functional capacity. Without *PEG11/RTL1*—an essential gene for preserving the internal integrity of fetal capillaries throughout gestation in eutherians [36]—marsupials may simply have been unable to exploit the full potential of the chorioallantoic placenta over a prolonged pregnancy. From this perspective, marsupials may have adopted a reproductive strategy optimized for short gestation, relying primarily on the yolk sac placenta despite retaining a latent capacity to form a chorioallantoic placenta. In contrast, the acquisition of both *PEG10* and *PEG11/RTL1* in the eutherian lineage provided the genetic foundation for maintaining a functional chorioallantoic placenta throughout long gestation. Acting together, these two viral genes were likely indispensable for the extended fetal growth characteristic of eutherian reproduction [18,19,20] (Table 2).

Building upon this idea—that differences in gene complement may have constrained the duration and functionality of the marsupial placenta—we next consider how these developmental disparities may have shaped the broader evolutionary trajectory of viral gene acquisition in therian mammals. If eutherians alone were capable of sustaining a long-acting chorioallantoic placenta, this would have fundamentally altered the selective and developmental environment in which viral genes could be domesticated. In line with the “placenta-driven evolution” hypothesis, the placenta may have served as a permissive site for the acquisition and domestication of virus-derived sequences, thereby facilitating repeated recruitment events. If this is the case, the effective duration of placental function—during which the tissue exists, remains transcriptionally active, and maintains relatively low DNA methylation—would directly influence the likelihood of viral gene acquisition. From this perspective, the striking contrast in the number of SIRH/RTL genes between eutherians—11 genes, including *PEG10*, *PEG11/RTL1*, and *SIRH3* through *SIRH11*—and marsupials, which possess only *PEG10* and *SIRH12* [24,101], may reflect these markedly different placental characteristics. A similar pattern is observed for the PNMA gene family, another metavirus-derived group: eutherians retain nearly twenty PNMA genes [99], whereas marsupials possess only a single member, *PNMA-MS1* [102]. These differences strongly suggest that the eutherian lineage experienced a substantially higher frequency of viral gene domestication.

The “placenta-driven evolution” hypothesis further highlights the importance of tissues characterized by reduced DNA methylation, because leaky transcription in such environments enables viral sequences to be expressed, evaluated, and potentially co-opted into functional roles in a relatively safe environment. Recent studies indicate that, unlike eutherians, marsupials do not undergo global DNA demethylation during preimplantation development [131]. Although marsupial placental tissues display lower methylation than embryonic tissues, the degree of hypomethylation is modest compared with that of eutherians [131]. This developmental difference may have further contributed to the disparity in viral gene acquisition between the two lineages. Taken together, these considerations suggest that the extended duration and epigenetic permissiveness of the eutherian placenta created a uniquely favorable environment for the recurrent acquisition and domestication of virus-derived genes. Consequently, viral gene domestication may have been tightly intertwined with the evolutionary diversification of placental form and function across therian and, more specifically, eutherian lineages.

## 4. Conclusions

The placenta is an evolutionarily novel organ that emerged in therian mammals. Although more than a thousand genes are thought to contribute, directly or indirectly, to placental development and function, a small number of metavirus- and retrovirus-derived genes have made disproportionately important contributions to this evolutionary innovation. Among these, ***PEG10***, the first domesticated gene acquired in the common therian ancestor, plays multiple critical roles in placental formation and maintenance. In contrast, ***PEG11/RTL1***, which was domesticated only in the eutherian lineage, is essential for the maintenance of placental internal vasculature, thereby enabling the prolonged gestation characteristic of eutherian mammals. ***SIRH7/LDOC1,*** another eutherian-specific gene, plays important roles of trophoblast differentiation and maturation, thereby facilitating the endocrine functions of the placenta during gestation. Together, these observations indicate that metaviral gene domestication played a central role in the emergence and subsequent elaboration of the placenta. In addition, **two *****syncytin***** genes**, domesticated from retroviruses in a lineage-specific manner within therians, play essential roles in the formation of syncytial layers surrounding fetal capillaries in the placenta.

These virus-derived genes were likely domesticated through a two-step evolutionary process: first, their retention by genetic drift (nearly neutral evolution) under conditions of relaxed epigenetic repression, such as low DNA methylation in extraembryonic tissues including the placenta and yolk sac, and second, their subsequent functional refinement by Darwinian natural selection.

Following the establishment of the placenta through the domestication of *PEG10*, the number of virus-derived genes increased markedly in therian lineages. This expansion was particularly pronounced in eutherians with prolonged gestation, exemplified by the domestication of *PEG11/RTL1*, in contrast to marsupials, which retain a short gestation period. Notably, all ten eutherian-specific SIRH/RTL genes examined to date play essential roles either in the placenta or in microglia, which originate from the yolk sac during early development. Together, these patterns imply that the placenta and yolk sac—and possibly other extraembryonic tissues—have acted as engines of mammalian evolution by facilitating the recurrent acquisition and functional integration of virus-derived genes. This evolutionary process can be conceptualized as **placenta-driven evolution**.

## Figures and Tables

**Table 1:** Brain functions of SIRH/RTL genes.

SIRH/RTL Genes	Official Name	Aliases	Conditions	Brain Related Phenotypes in Mice	Related Human Disorders
*PEG10*	*PEG10*	*RTL2*, *SIRH1*	Over	N.D.	Angelman syndrome (AS)?
					Amyotrophic lateral sclerosis (ALS)?
*PEG11*	*RTL1*	*SIRH2*	Over	Reduced activity and memory, increased anxiety	Kagami-Ogata syndrome (KOS14)
			KO	Reduced activity, increased anxiety	Temple syndrome (TS14)
*SIRH3*	*RTL6*	*LDOC1L*	KI, KO	Defense against bacterial LPS in the brain	Inflammatory disease?
*SIRH4*	*RTL8C*	*CXX1C*	Red, KO	Depression-like behavior, late-onset obesity, abnormal maternal behavior	Prader–Willi syndrome (PWS)?
*SIRH5*	*RTL8A*	*CXX1A*	Red, KO		
*SIRH6*	*RTL8B*	*CXX1B*	Red, KO		
*SIRH7*	*LDOC1*	*RTL7*	KO	Abnormal maternal behavior	
*SIRH8*	*RTL5*	*RGAG4*	KI, KO	Defense against viral dsRNA and non-methylated DNA in the brain	Inflammatory disease?
*SIRH9*	*RTL3*	*ZCCHC5*		N.D.	
*SIRH10*	*RTL9*	*RGAG1*	KI, KO	Defense against fungal cell wall (zymosan) in the brain	Inflammatory disease?
*SIRH11*	*RTL4*	*ZCCHC16*	KI, KO	Increased impulsivity, Inadaptability to a new environment, reduced short spatial memory, noradrenaline response	Autism spectrum disorder (ASD)

KI: knock-in; KO: knockout; Over: overexpression; Red: reduced expression (DKO of triplet genes). Overexpression of PEG10 is induced by lack of UBE3A and/or UBQLN2 [62,63]. ND: not determined.

**Table 2:** Cross-species comparison of *PEG10*, *PEG11/RTL1* and *Syncytin* functioning in mouse and human placenta.

Category	Mouse (Mus Musculus)	Human (Homo Sapiens)	Notes
**Placental** **type and architecture**	Hemochorial placenta with labyrinth (LA), spondiotrophoblast (ST) and trophoblast giant cell (TGC) layers. LA composed of fetal endothelium, SynT-I/II and s-TGC, plus junctional zone [2,15,113]	Hemochorial villous placenta with VCT, STB, and EVT [2,15,113]	Both mice and humans possess hemochorial placentas (ancestral type). These architectures are distinct (labyrinth versus. villi). However, the STB appears to correspond to SynT-I/II [113]. Functional similarities are also observed between the VCT and trophoblast progenitor cells in LA as proliferative progenitors [45,114], as well as between the EVT and TGC in their ability to invade the maternal decidua [115,116].
***PEG10***			
***PEG10*** **-origin**	Metavirus-derived, paternally expressed imprinted gene [28]	Metavirus-derived, paternally expressed imprinted gene [25]	Highly conserved origin and imprinting [28]
***PEG10*** **—placental expression**	Trophoblast cell lineage [47,50]	Trophoblast cell lineage [117,118,119]	*PEG10* shows highly expression in both mouse and human trophoblast cells, although trophoblast cell types are different (equivalent cell types are inferred, not identical)
***PEG10*** **—functional evidence**	Loss-of-function causes severe placental defects and embryonic lethality; impaired trophoblast differentiation and placental organization [28,44,47]	Direct genetic evidence limited; expression and association studies implicate roles in preeclampsia and placental development [117,118,119]	Multiple *PEG10* mutant studies in mice strongly suggest that *PEG10* is one of the key regulatory genes for trophoblast differentiation in not only mouse but also all (eu)therian mammals including human [120,121]
***PEG11/RTL1***			
***PEG11/RTL1—*** **origin**	Metavirus-derived, paternally expressed imprinted gene [36,48,49]	Metavirus-derived, paternally expressed imprinted gene [37]	Highly conserved origin and imprinting
***PEG11/RTL1*** **—placental expression**	Strongly expressed in fetal endothelial cells and Syn-TII within the labyrinth vasculature [36,50]	Expressed in fetal endothelial cells and associated pericytes within chorionic villi [122]	One of the clearest mouse–human correspondences in placental vasculature
***PEG11/RTL1*** **—functional evidence**	Both loss and overproduction cause defects of fetal capillaries at the feto–maternal interface similar to human KOS14 and TS14 [36,68,123]	Human data include expression analyses and relevance to imprinting disorders, KOS14 and TS14 [37,67,68]	Endothelial localization is a key conserved feature
***Syncytin***			
***Syncytin*** **—gene set & origin**	Mouse have rodent-specific *syncytin-A* and *syncytin-B* genes derived from rodent-specific retroviral *ENV* genes [35]	Humans (and other primates) possess *syncytin-1* and *syncytin-2* derived from *primate-specific* retroviral *ENV* genes [31,32,33,34,35]	The fusion function is conserved even when the captured *ENV* genes differ between humans and mice. Other *syncytins* and *syncytin*-related genes in other therian lineages have been identified [124,125,126,127,128,129,130]
**Syncytin—functional evidence**	Syncitin genes mediate trophoblast cell–cell fusion to generate syncytiotrophoblasts; Both *syncytin-A* and *B* are essential role in placentation and the latter also in immunosuppression [58,59].	*Syncytin-1* and *2* are identified as fusogenic and implicated in trophoblast fusion and the latter also in immunosuppression [31,32,33,34,35,60]	

## Data Availability

No new data were created or analyzed in this study.

## Abbreviations

The following abbreviations are used in this manuscript:

ERV	Endogenous retrovirus
LDOC1	Leucine-zipper downregulated in cancer 1
PEG10	Paternally expressed 10
PEG11	Paternally expressed 11
PNMA	Paraneoplastic Ma antigen
RTL	Retrotransposon Gag-like
SIRH	Sushi-ichi retrotransposon homolog
